# Humanised monoclonal antibodies neutralise pertussis toxin by receptor blockade and reduced retrograde trafficking

**DOI:** 10.1111/cmi.12948

**Published:** 2018-09-23

**Authors:** Edith Acquaye‐Seedah, Yimin Huang, Jamie N. Sutherland, Andrea M. DiVenere, Jennifer A. Maynard

**Affiliations:** ^1^ Department of Biochemistry The University of Texas at Austin Austin Texas; ^2^ Department of Cell and Molecular Biology The University of Texas at Austin Austin Texas; ^3^ Department of Chemical Engineering The University of Texas at Austin Austin Texas

**Keywords:** antibody engineering, Bordetella, neutralisation, passive immunotherapy, toxins

## Abstract

Pertussis toxin (PTx) is a major protective antigen produced by *Bordetella pertussis* that is included in all current acellular vaccines. Of several well‐characterized monoclonal antibodies binding this toxin, the humanised hu1B7 and hu11E6 antibodies are highly protective in multiple in vitro and in vivo assays. In this study, we determine the molecular mechanisms of protection mediated by these antibodies. Neither antibody directly binds the *B. pertussis* bacterium nor supports antibody‐dependent complement cytotoxicity. Both antibodies, either individually or as a cocktail, form multivalent complexes with soluble PTx that bind the FcγRIIb receptor more tightly than antibody alone, suggesting that the antibodies may accelerate PTx clearance via immune complex formation. However, a receptor binding assay and cellular imaging indicate that the main mechanism used by hu11E6 is competitive inhibition of PTx binding to its cellular receptor. In contrast, the main hu1B7 neutralising mechanism appears to be inhibition of PTx internalisation and retrograde trafficking. We assessed the effects of hu1B7 on PTx retrograde trafficking in CHO‐K1 cells using quantitative immunofluorescence microscopy. In the absence of hu1B7 or after incubation with an isotype control antibody, PTx colocalizes to organelles in a manner consistent with retrograde transport. However, after preincubation with hu1B7, PTx appears restricted to the membrane surface with colocalization to organelles associated with retrograde transport significantly reduced. Together, these data support a model whereby hu11E6 and hu1B7 interfere with PTx receptor binding and PTx retrograde trafficking, respectively.

## 1 INTRODUCTION

Despite widespread vaccine availability, severe infection with *Bordetella pertussis* continues to afflict infants and young children, often with serious consequences (Mattoo & Cherry, 2005). More troubling, disease rates have been rising steadily over the past few decades, to a 50‐year high of 48,000 cases in 2012 (Lavine, King, Andreasen, & Bjornstad, 2013; Tanaka et al., 2003). Vaccination prevents the severe manifestations of disease but has been unable to eliminate subclinical infection (Lavine, King, & Bjornstad, 2011). In fact, the temporal decline in postvaccination immunity has altered disease demographics such that adults and adolescents now constitute the dominant reservoir (Senzilet et al., 2001). To protect the most vulnerable populations, booster vaccinations have been recommended for adolescents, families of infants, and, more recently, pregnant women. In order to design improved vaccines, it is crucial to understand mechanisms of protective immunity against pertussis, especially those mediated by antibodies recognising vaccine components.

A challenge in pertussis vaccine development is that *B. pertussis* produces multiple virulence factors (Robbins et al., 1998; Robbins et al., 2009). Of these, bacterial attachment to eukaryotic cells is facilitated by filamentous haemagglutinin (Fha), fimbriae, and pertactin (Tuomanen & Weiss, 1985; van't Wout et al., 1992), whereas the pertussis toxin (PTx) and adenylate cyclase toxins interfere with normal host immune responses (Smith, Guzman, & Walker, 2001). Current acellular whooping cough vaccines contain chemically detoxified PTx plus one to four additional virulence factors. Although there is no accepted immune correlate of protection, PTx is directly responsible for leukocytosis, which is in turn predictive of disease severity and clinical outcome (Carbonetti, 2016; Murray et al., 2013).

PTx is a 105‐kDa member of the AB_5_ toxin class, which includes cholera toxin, the shiga toxins, and Escherichia coli heat labile enterotoxin, among others (Beddoe, Paton, Le Nours, Rossjohn, & Paton, 2010). These toxins all include a catalytically active A subunit (termed S1 in PTx) and a receptor binding, pentameric B subunit. During toxin assembly and secretion, the toxin is transiently associated with the bacterial surface (Farizo, Fiddner, Cheung, & Burns, 2002). Upon binding to terminally sialylated cell surface receptors, PTx is endocytosed into early endosomes, followed by retrograde transport to the Golgi and then to the endoplasmic reticulum (ER; Plaut & Carbonetti, 2008). Here, reduction of a disulfide bond in S1 and ATP‐induced conformational changes in the B subunit result in release of S1 and transport of unfolded S1 from ER into the eukaryotic cytoplasm (Plaut, Scanlon, Taylor, Teter, & Carbonetti, 2016). In the cytoplasm, S1 catalyses the ADP‐ribosylation of G_i/o_ proteins, disrupting G‐protein signalling (Katada, Tamura, & Ui, 1983; Katada & Ui, 1982) and compromising neutrophil/macrophage activities (Kirimanjeswara, Agosto, Kennett, Bjornstad, & Harvill, 2005; Meade, Kind, Ewell, McGrath, & Manclark, 1984; Meade, Kind, & Manclark, 1984; Schaeffer & Weiss, 2001).

PTx exerts a range of effects in cultured cells and in animal models. In CHO‐K1 cell lines, it disrupts contact inhibition, resulting in a clustered growth morphology (Hewlett, Sauer, Myers, Cowell, & Guerrant, 1983), which is used as a convenient in vitro assay for toxin activity. In animals and humans, the most noticeable PTx effect is leukocytosis, as the circulating white blood cell count can be elevated many‐fold (Carbonetti, 2016). Consequences of leukocytosis include inhibited neutrophil recruitment to the infection site (Kirimanjeswara et al., 2005) and altered mononuclear phagocyte circulation, which together reduce bacterial killing (Schaeffer & Weiss, 2001). PTx also promotes *B. pertussis* colonisation by reducing expression of pro‐inflammatory cytokines from alveolar macrophages and epithelial cells and inhibiting the activity of chemokines important for recruiting neutrophils and macrophages to the site of infection (Andreasen & Carbonetti, 2008; Rollins, 1997).

Due to PTx's immunogenicity and role as a major protective antigen, many anti‐PTx antibodies have been generated and characterized for their ability to protect in in vitro and in vivo assays. Two of these, the murine m1B7 and m11E6 antibodies, are potently neutralising all relevant assays (Sato, Ito, Chiba, & Sato, 1984; Sato & Sato, 1990). Humanised versions of these antibodies, hu1B7 and hu11E6, retained low nanomolar affinities for PTx, prevented leukocytosis when delivered individually in a mouse infection model (Nguyen et al., 2015), and exhibited synergy when coadministered to mice in a toxin challenge model (Wagner, Wang, Bui, & Maynard, 2016). The combination of both humanised antibodies dramatically halted leukocytosis when administered to adolescent baboons 3 days after experimental infection with *B. pertussis* (Nguyen et al., 2015).

In this study, we determine the mechanisms by which the hu11E6 and hu1B7 antibodies inhibit PTx cellular toxicity. Prior work defined the hu1B7 epitope with amino acid‐level resolution (Kowalsky et al., 2015; Sutherland & Maynard, 2009), revealing an epitope that is dominated by S1 but appearing to bridge the subunit interface to weakly engage the hetero‐pentameric PTx B subunit (Sutherland & Maynard, 2009). The m11E6 epitope has been determined to include the S2/3 subunits near the receptor‐binding site (Sato, Sato, Ito, & Ohishi, 1987). Neutralising antibodies can potentially interfere with any of the steps involved in PTx secretion, receptor binding, and intracellular trafficking (Wagner & Maynard, 2018). Here, we explore these antibodies' effects on each step in PTx intoxication to determine the molecular mechanisms by which they interfere with PTx function.

## 2 RESULTS

### 2.1 Neither antibody directly binds *Bordetella pertussis* nor mediates complement‐dependent bactericidal activity

**Figure 1:**
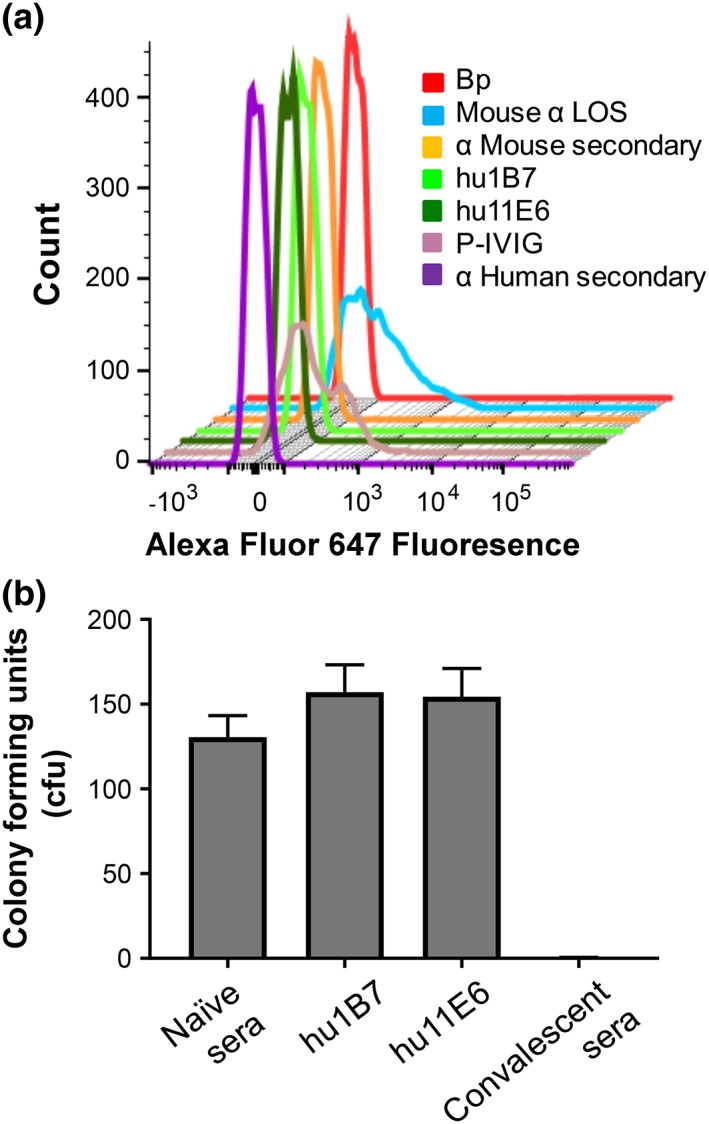
Neither hu1B7 nor hu11E6 exert direct bactericidal activities. (a) Antibody binding to *Bordetella pertussis* cells, monitored by flow cytometry. *B. pertussis* TohamaI cells were grown under conditions supporting expression of pertussis toxin and other BvgAS‐regulated virulence factors. Cells were washed with Stainer–Scholte media (SSM), incubated with 10 μg ml^−1^ antibody in SSM, followed by anti‐mouse or anti‐human IgG‐Alexa‐647, before a final wash, and scan on a BD LSR Fortessa. Positive controls include a mouse anti‐Bp LOS antibody and human P‐IVIG. (b) Complement bactericidal assay. *B. pertussis* TohamaI cells in SSM were incubated with naïve baboon sera and 25 μg ml^−1^ purified human antibodies for 1 hr; dilutions were plated in BG plates to quantify bacterial colony‐forming units (cfu). The naïve and positive control convalescent baboon sera were collected in our previous project (Nguyen et al., 2015). Neither the anti‐LOS antibody nor P‐IVIG reduced cfu counts (data not shown). The experiment was performed twice with duplicates, whereas the cfu measurements were performed in triplicate by bacterial plating. Shown are the average and standard deviation from one representative experiment

Antibodies against surface exposed antigens on *B. pertussis* have been reported to promote phagocytosis and/or complement mediated cell lysis (Aase et al., 2011; Hellwig, Rodriguez, Berbers, van de Winkel, & Mooi, 2003). Moreover, treatment of mice with hu1B7 or hu11E6 before bacterial infection accelerated bacterial clearance (Nguyen et al., 2015; Sato & Sato, 1990). Because PTx is transiently associated with the bacterial surface during export through dedicated Type IV secretion machinery (Farizo et al., 2002), we first tested whether hu1B7 can exert direct antibacterial effects by binding cells and recruiting complement. Antibody binding to *B. pertussis* was monitored by flow cytometry after incubating 10 μg ml^−1^ hu1B7, hu11E6, anti‐Bp LOS (Santa Cruz Biotech), human P‐IVIG, or a human IgG1 isotype control antibody, followed by detection with a fluorescently labelled secondary antibody. The positive control anti‐LOS and human P‐IVIG both showed strong binding to the bacterial cells. In contrast, neither the isotype control antibody nor the anti‐PTx antibodies exhibited detectable binding (Figure 1a).

We performed a second experiment, in which *B. pertussis* cells were preincubated with the same antibodies and then sera to monitor complement‐dependent lysis. Here, early log phase bacteria were equilibrated with 25 μg ml^−1^ antibody in SSM growth media, before adding naïve or convalescent baboon sera to a final concentration of 10% and enumerating surviving bacteria by colony counts. We observed a high killing rate for bacteria incubated with positive control serum from a convalescent animal from our previous study (Nguyen et al., 2015; Figure 1b). By contrast, we did not observe any killing after incubation with hu1B7, hu11E6, or an isotype control antibody. These results are in agreement with previous studies using polyclonal serum containing antibodies to PTx, Fha, and pertactin, suggesting that opsonophagocytic activity is due primarily to anti‐pertactin antibodies (Gotto et al., 1993; Hellwig et al., 2003) and possibly anti‐Fha antibodies (Aase et al., 2011). Similarly, the ability of hu1B7 and hu11E6 to reduce bacterial load in animal models does not appear to result from direct bactericidal effects but indirectly by protecting innate immune cells from PTx activities (Nguyen et al., 2015; Sato & Sato, 1990).

### 2.2 Antibody–PTx immune complexes bind FcγRIIb with high affinity

The antibody Fc and its effector functions are generally not required to neutralise secreted toxins (Bournazos, DiLillo, & Ravetch, 2015); however, this domain confers extended antibody in vivo half‐life and can support accelerated toxin clearance by formation of small immune complexes (Ganesan et al., 2012; Montero‐Julian, Klein, Gautherot, & Brailly, 1995). These are formed by two or more antibodies binding simultaneously to one toxin molecule and are rapidly cleared via the increased avidity of multimerized Fc for Fcγ receptors (Montero‐Julian et al., 1995). Hu11E6 binds an epitope present in each of the homologous S2 and S3 subunits, which means two high‐affinity epitopes are present per toxin molecule (Sato et al., 1987). Hu1B7 binds a single epitope on the S1 subunit with weak binding to the S4 subunit on the B‐oligomer (Sutherland & Maynard, 2009). Because each PTx molecule includes two S4 subunits, this provides a basis for bivalent hu1B7 antibodies to generate immune complexes.

**Figure 2:**
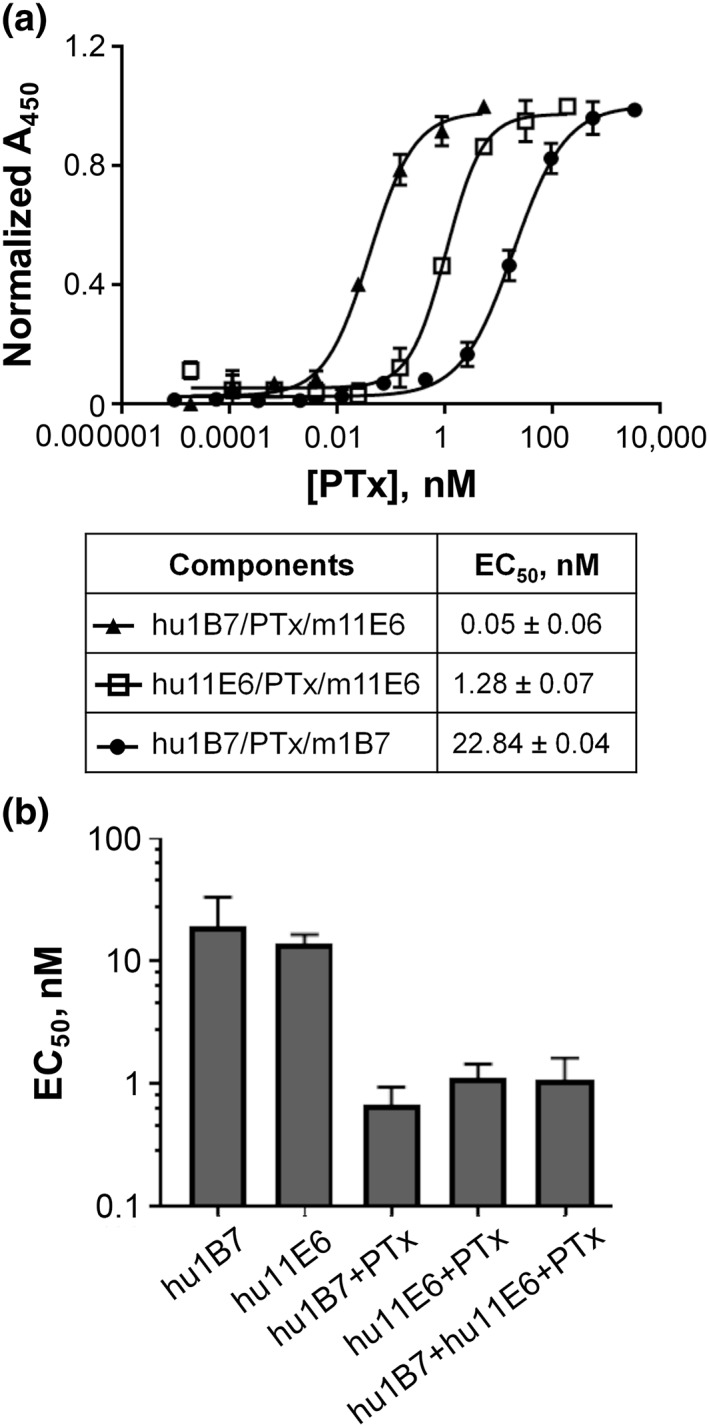
Hu1B7 and hu11E6 support formation of multivalent immune complexes in vitro. (a) Antibody hu1B7 and hu11E6 each recognize two binding epitopes on pertussis toxin (PTx). The humanised antibody hu1B7 or hu11E6 was coated onto ELISA plates at 1 μg ml^−1^ and blocked. PTx was serially diluted and added to these wells, followed by m1B7 or m11E6 at 1 μg ml^−1^ and secondary goat anti‐mouse HRP antibody, TMB substrate for visualisation, and data collection at 450 nm. Signal was normalised by dividing all data points by the highest observed absorbance value for each dataset; the EC_50_ values were determined from four‐parameter non‐linear fits using GraphPad Prism 5. Error bars represent standard error for duplicate samples in the experiment; the experiment was performed twice with similar results. (b) Binding of antibody–PTx complexes to FcγRIIb. Human FcγRIIb was coated onto ELISA plates at 2 μg ml^−1^. In a separate blocked plate, 20 nM PTx was preincubated with serially diluted hu1B7, hu11E6, or an equimolar mixture of hu1B7 and hu11E6 such that the initial total antibody: PTx molar ratio was 15:1, and transferred to the coated plate. Bound antibody was detected with anti‐human‐Cκ‐HRP, and EC_50_ values were determined from four‐parameter non‐linear fits using GraphPad Prism 5. Average EC_50_ values from duplicate samples are reported; the experiment was performed three times with similar results

To determine whether PTx can simultaneously engage multiple 1B7 antibodies to form small immune complexes, we devised an ELISA scheme with hu1B7 and m1B7 to mimic this process. As a control, antibody hu1B7 was coated on the plate and then incubated with PTx and finally with a second m11E6 antibody that binds to an epitope that does not overlap with 1B7 epitopes. This arrangement showed strong binding at low PTx concentrations (EC_50_ ~0.05 nM; Figure 2a). As a second control, coated hu11E6 was able to bind PTx that could in turn support binding by a second m11E6 antibody, albeit with reduced sensitivity due to the weaker affinity of the 11E6 as compared with 1B7 antibodies (EC_50_ ~1 nM PTx; Figure 2a). Binding of PTx to coated hu1B7 followed by the m1B7 antibody, exhibited binding only at higher PTx concentrations (EC_50_ ~23 nM; Figure 2a). These data support the presence of a lower affinity secondary binding site for 1B7 on PTx and the potential for both hu11E6 and hu1B7 to form immune complexes.

The clearance of small immune complexes is facilitated by the increased avidity of the multimeric complex for low‐affinity Fc receptors as compared with the monomeric antibody (Daeron, 1997). In mice, FcγRIIb has been implicated in the clearance of such small immune complexes (Ganesan et al., 2012). We therefore determined whether mixtures of PTx–hu11E6 and PTx–hu1B7 bind human FcγRIIb more strongly than the individual antibodies, suggesting that immune complexes form. When a 15‐fold molar excess of hu11E6 was preincubated with PTx, strong binding to immobilised FcγRIIb was observed by ELISA. In contrast, when hu11E6 or hu1B7 was added at the same concentration but without PTx, weak binding was observed (Figure 2b). When a 15‐fold molar excess of each antibody was preincubated with 20 nM PTx, stronger binding to FcγRIIb was observed than for the same concentration of antibody without PTx (>100‐fold improved ELISA sensitivity; Figure 2b). These data support a potential role for hu1B7 and hu11E6 in FcγRIIb‐mediated PTx removal.

### 2.3 Antibody hu11E6 blocks PTx binding to the cellular receptor

**Figure 3:**
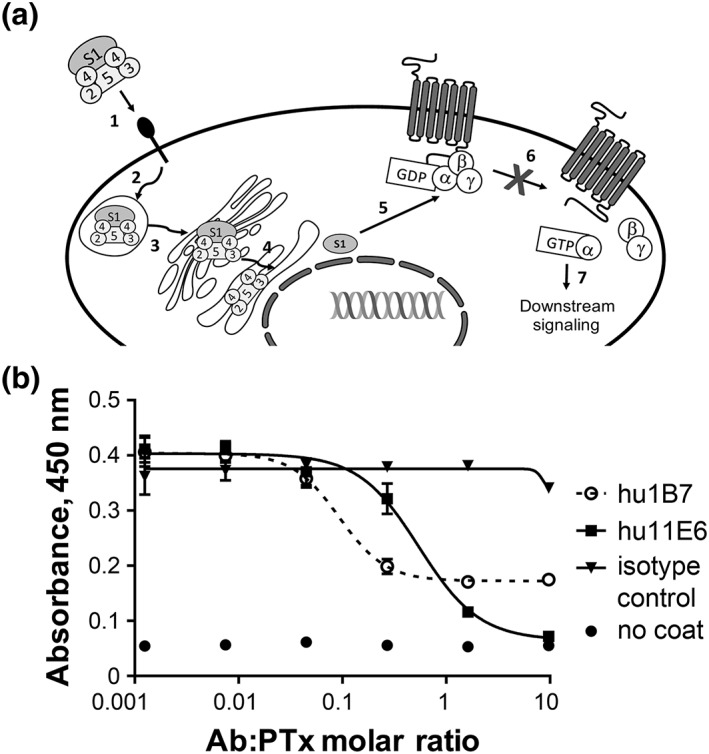
Antibody hu11E6 blocks pertussis toxin (PTx) binding to cellular receptors. (a) The presumptive PTx intracellular trafficking path. After the PTx heteropentameric B subunit binds terminally sialylated cellular receptors (1), PTx undergoes receptor‐mediated endocytosis into early endosomes (2), followed by retrograde transport to the Golgi (3), and then the endoplasmic reticulum (4). Within the endoplasmic reticulum, the presence of ATP and a reducing environment is thought to facilitate unfolding and release of the S1 subunit, which is translocated to the cytoplasm, likely through the ERAD Sec61 pore (5). In the cytoplasm, the S1 subunit refolds and catalyses transfer of the ADP‐ribosyl moiety from NAD to membrane associated inhibitory and olfactory G‐alpha proteins (6). The inactivated G‐proteins are then unable to mediate downstream signalling events (7). (b) Effect of antibodies on PTx binding to the model terminally sialylated receptor, fetuin. Serially diluted humanised antibodies were incubated with 0.2 μg ml^−1^ PTx, and the complex was added to fetuin‐coated and blocked ELISA plates. The plates were washed, and PTx was detected with a murine anti‐PTx antibody cocktail, anti‐mouse‐IgG‐HRP, and TMB substrate, with signal at 450 nm recorded. Shown are the average signal and standard error from one experiment; the experiment was performed three times with similar results

PTx undergoes a series of steps before accessing its ultimate target in the eukaryotic cytoplasm, many of which could be susceptible to antibody‐mediated inhibition (Figure 3a). Antibodies recognising the AB‐family toxins have been shown to neutralise by mechanisms including blocking receptor‐binding sites on the toxin (Orth et al., 2014), preventing conformational changes essential for toxin activity (Oganesyan et al., 2014), and altering intracellular trafficking (Yermakova, Klokk, Cole, Sandvig, & Mantis, 2014). We first determined whether hu1B7 or hu11E6 could disrupt the initial step in toxin internalisation by interfering with PTx binding to a model receptor. Antibody hu11E6 binds an epitope in the S2 and S3 subunits of the B‐oligomer, which also contains the receptor‐binding epitope. This antibody has been proposed to interfere with PTx binding to receptors (Sato et al., 1987).

For this ELISA, fetuin was used as a model terminally sialylated receptor for PTx (Acquaye‐Seedah et al., 2018; Kenimer et al., 1989) and coated on ELISA plates. Separately, PTx or antibody plus PTx (an initial antibody molar excess of 10:1) were combined in a fixed volume and allowed to equilibrate before transfer to fetuin‐coated and blocked ELISA wells. Fetuin‐captured PTx was detected with a cocktail of mouse anti‐PTx antibodies followed by an anti‐mouse secondary antibody. When present at molar ratios over one, antibody hu11E6 completely inhibited PTx binding to fetuin, whereas no inhibition was observed for an isotype control antibody at ratios up to 10. Surprisingly, we also observed partial inhibition of fetuin binding by hu1B7 (Figure 3b). Because hu1B7's effect on receptor binding was equivocal and insufficient to fully explain its potent neutralising effects, we next sought to determine whether hu1B7 could also alter intracellular steps.

### 2.4 Antibodies hu1B7 and hu11E6 can each bind receptor‐bound PTx

**Figure 4:**
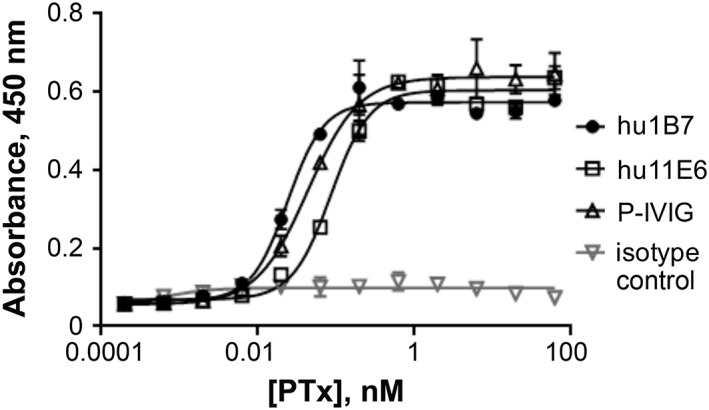
Antibodies hu1B7 and hu11E6 bind receptor‐bound pertussis toxin (PTx). Serially diluted PTx was added to fetuin‐coated and blocked ELISA plates. The indicated antibodies were added at 1 μg ml^−1^, followed by secondary goat anti‐human HRP antibody and TMB substrate, with signal at 450 nm recorded. Shown are the mean and standard error from one experiment; the experiment was performed three times with similar results

In order for an antibody to directly alter PTx intracellular trafficking, it must first bind receptor‐bound PTx. Again using fetuin as a model receptor, we developed an ELISA to monitor antibody binding to immobilised PTx–fetuin complexes. Fetuin was first coated onto ELISA plates, PTx was added and allowed to equilibrate, and then antibody dilutions were added and detected with anti‐human secondary antibody. Antibodies hu1B7, hu11E6, and P‐IVIG all showed strong binding to PTx–fetuin complexes, whereas an isotype control antibody or omission of PTx resulted in no binding (Figure 4). Similar results were observed for binding of the murine antibodies (m1B7 and m11E6) to PTx captured by a different model immobilised receptor, transferrin (Figure S1). Because there are two receptor‐binding sites on PTx, one seems to be engaged in binding receptor, leaving the other available for hu11E6 binding.

After binding to a cellular receptor, PTx is endocytosed and follows a path of retrograde transport, travelling through the early/recycling endosomes to the Golgi and then to the ER (Plaut & Carbonetti, 2008). Here, the presence of a reducing potential and ATP reduces a disulfide bond in the S1 subunit, facilitating its release from the PTx B subunit, and transports out of the ER into the cytosol (Plaut et al., 2016; Figure 3a). In order for hu1B7 to directly influence PTx in any of these compartments, the antibody would need to retain high‐affinity binding in the unique biochemical conditions corresponding to each subcellular compartment. We therefore monitored PTx–hu1B7 binding under different pH and temperature conditions using indirect ELISAs, to mimic conditions of the subcellular compartments. We observed that at 37°C, decreasing the pH from 7.2 to 5.0 had minimal effect on hu1B7 binding to PTx, suggesting that the complex persists in the acidic endosome. At higher temperatures, binding was reduced only at the lowest pH tested, 5.0 (Table S1).

### 2.5 Hu1B7 interferes with PTx retrograde trafficking

Next, we directly assessed the effects of hu1B7 on PTx intracellular trafficking of PTx. Retrograde transport of PTx has been observed in CHO‐K1 epithelial, mouse AMJ2‐C8 alveolar macrophages, and human A549 lung epithelial cell lines (Plaut & Carbonetti, 2008). ADP‐ribosylated G‐proteins have been detected 1 hr after the addition of PTx to cultured cells, indicating that PTx is internalised and arrives in the cytoplasm within this time frame (Xu & Barbieri, 1995). In other studies, tyrosine sulfated PTx–S1, a modification occurring in the Golgi, could be detected within 2 hr, whereas N‐linked glycosylation, a modification occurring in the ER, could be detected within 3 hr (Plaut & Carbonetti, 2008).

**Figure 5:**
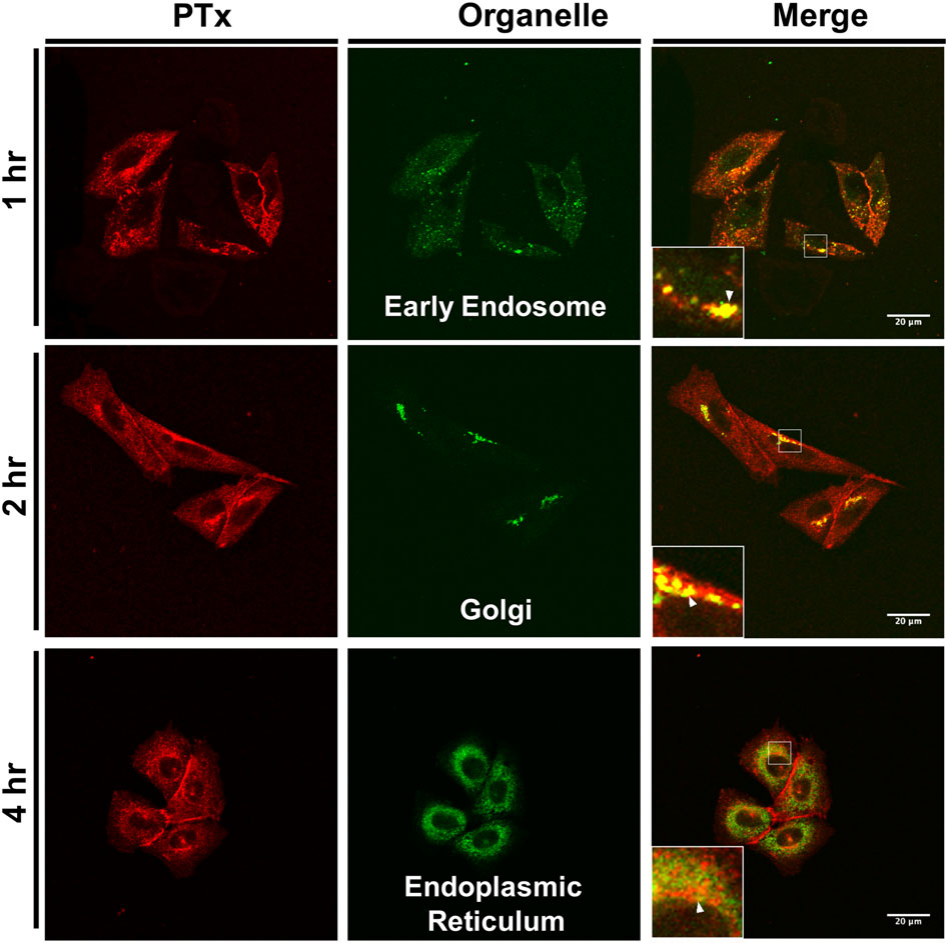
Pertussis toxin (PTx) retrograde trafficking in CHO cells. CHO cells were grown on cover slides and incubated with 1 nM PTx for varying times (60, 120, or 240 min). At the indicated time points, cells were fixed and stained. PTx was detected with a cocktail of mouse anti‐PTx antibodies followed by anti‐mouse IgG‐Cy5 (red), while simultaneously staining for a single organelle (green). CHO cells incubated with PTx for 1 hr were costained with rabbit antibodies recognising the EEA1 early endosome marker, whereas those incubated with PTx for 2 hr were costained with rabbit antibodies recognising the Golgin‐97, and those incubated with PTx for 4 hr were costained with rabbit antibodies recognising the endoplasmic reticulum‐resident protein disulfide isomerase. Each rabbit antibody was followed by goat anti‐rabbit IgG‐AF488. Data from at least 30–40 cells were collected on a confocal microscope; the white arrowhead in the inset indicates colocalization between PTx (red) and organelles (green). Images shown are representative of at least three independent experiments. Scale bar, 20 μm

We chose immunofluorescence microscopy to monitor PTx transport as a technically simpler strategy than the modified PTx approach used in prior reports (Plaut & Carbonetti, 2008). After growing CHO cells on coverslips to 50–70% confluency, we adjusted the media to include 1 or 10 nM PTx. Although this concentration is considerably higher than required for the CHO cell clustering assay, pilot experiments indicated that it was necessary for subsequent visualisation of PTx. We allowed the cells to incubate with PTx for varying times (60–240 min) before fixing and staining with antibodies to detect PTx or a specific organelle. Subsequent immunofluorescence microscopy revealed colocalization of PTx with EEA1‐positive early endosomes 60 min after PTx addition, with the Golgin97‐positive trans‐Golgi network at 120 min and with the ER resident enzyme, protein disulfide isomerase at 4 hr (Figure 5). These results are consistent with the previously observed kinetics of PTx retrograde trafficking (Plaut & Carbonetti, 2008).

We next determined the impact of antibodies hu11E6 and hu1B7 on PTx endocytosis and retrograde trafficking. Prior experiments indicated the PTx‐induced CHO cell clustering morphology is completely neutralised by a 1,000‐fold molar excess of hu11E6 or >5,000‐fold molar excess of hu1B7 in the presence of PTx (Acquaye‐Seedah et al., 2018; Wagner et al., 2016). We therefore preincubated 1 nM PTx with a 10,000‐fold molar excess hu1B7 or 10 nM PTx with a 1,000‐fold molar excess of hu11E6 before adding the complex to CHO cells grown on coverslips. Based on the measured hu1B7 affinity of 0.7 nM and hu11E6 affinity of 2.3 nM (Nguyen et al., 2015) and the definition of an equilibrium dissociation constant (Equation (1)), these conditions are expected to result in complexation of >99.99% of the PTx before equilibrium is disturbed by the presence of the cellular receptor on CHO cells.

(1) $$K_{D}=\left({\left[\mathrm{Ab}\right]}_{o}-{\left[\text{complex}\right]}_{\mathrm{eq}}\right)*\left({\left[\mathrm{PTx}\right]}_{o}-{\left[\text{complex}\right]}_{\mathrm{eq}}\right){\left[\text{complex}\right]}_{\mathrm{eq}},$$

where [Ab]_o_ indicates the initial concentration of antibody (10 μM), whereas [PTx]_o_ indicates the initial concentration of PTx (1 or 10 nM), and [complex] indicates the concentration of PTx–antibody complex at equilibrium.

**Figure 6:**
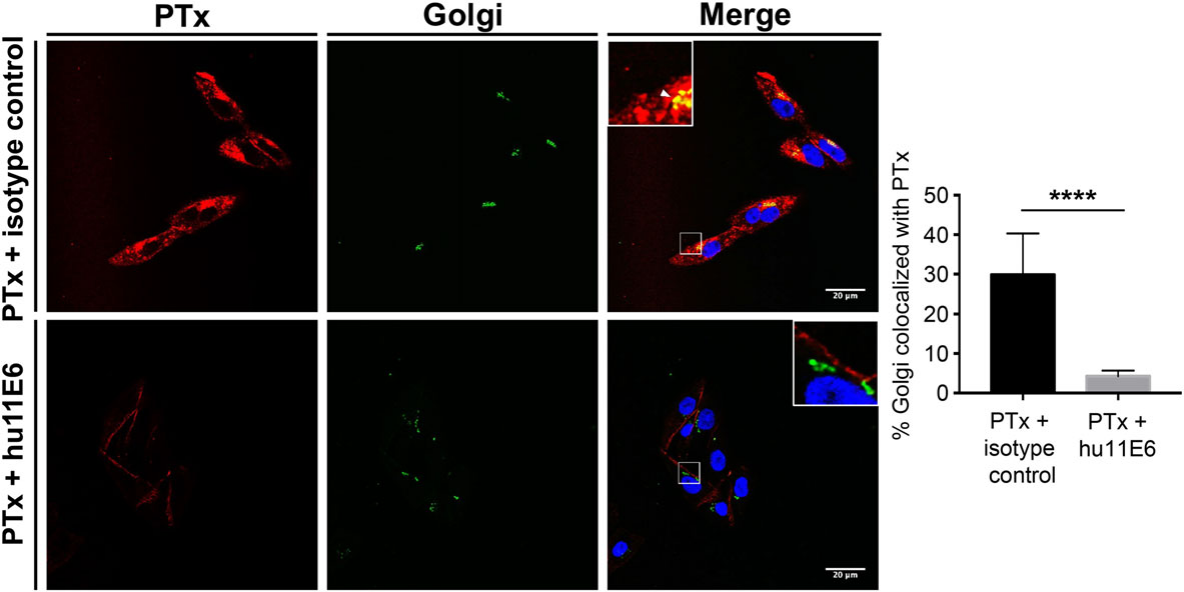
Hu11E6 reduces pertussis toxin (PTx) internalisation into CHO cells. CHO cells were grown on cover slides and incubated with 10 nM PTx pre‐equilibrated with a 1,000‐fold molar excess of hu11E6 antibody. After 2 hr, cells were fixed and stained with a cocktail of mouse anti‐PTx antibodies followed by goat anti‐mouse IgG‐Cy5 to detect PTx (red). The Golgi were stained with rabbit anti‐Golgin‐97 antibodies, followed by goat anti‐rabbit IgG‐AF488 (green), whereas the nuclei were labelled with 4,6‐diamidino‐2‐phenylindole (blue). The white arrowhead in the inset indicates colocalization between PTx (red) and organelles (green). The percent of Golgi pixels colocalizing with PTx pixels was determined using JACoP plugin for ImageJ. Shown are the mean and standard deviation of at least 20–30 cells from seven independent images; the isotype and hu11E6 treatments were performed at the same time to facilitate comparison. Results shown are representative of three independent experiments; **** p <0.0001. Scale bar, 20 μm

Immunofluorescence microscopy was then used to visualise PTx with the same timing while costaining for the same organelles conditions as above. In the presence of a 1,000‐fold molar excess of an isotype control antibody, ~30% of the labelled Golgi contained PTx, whereas in the presence of hu11E6, a very low PTx signal was observed at the cell surface, and just ~4% of Golgi contained PTx (Figure 6). This is consistent with a mechanism by which hu11E6 effectively blocks the PTx–receptor interaction.

**Figure 7:**
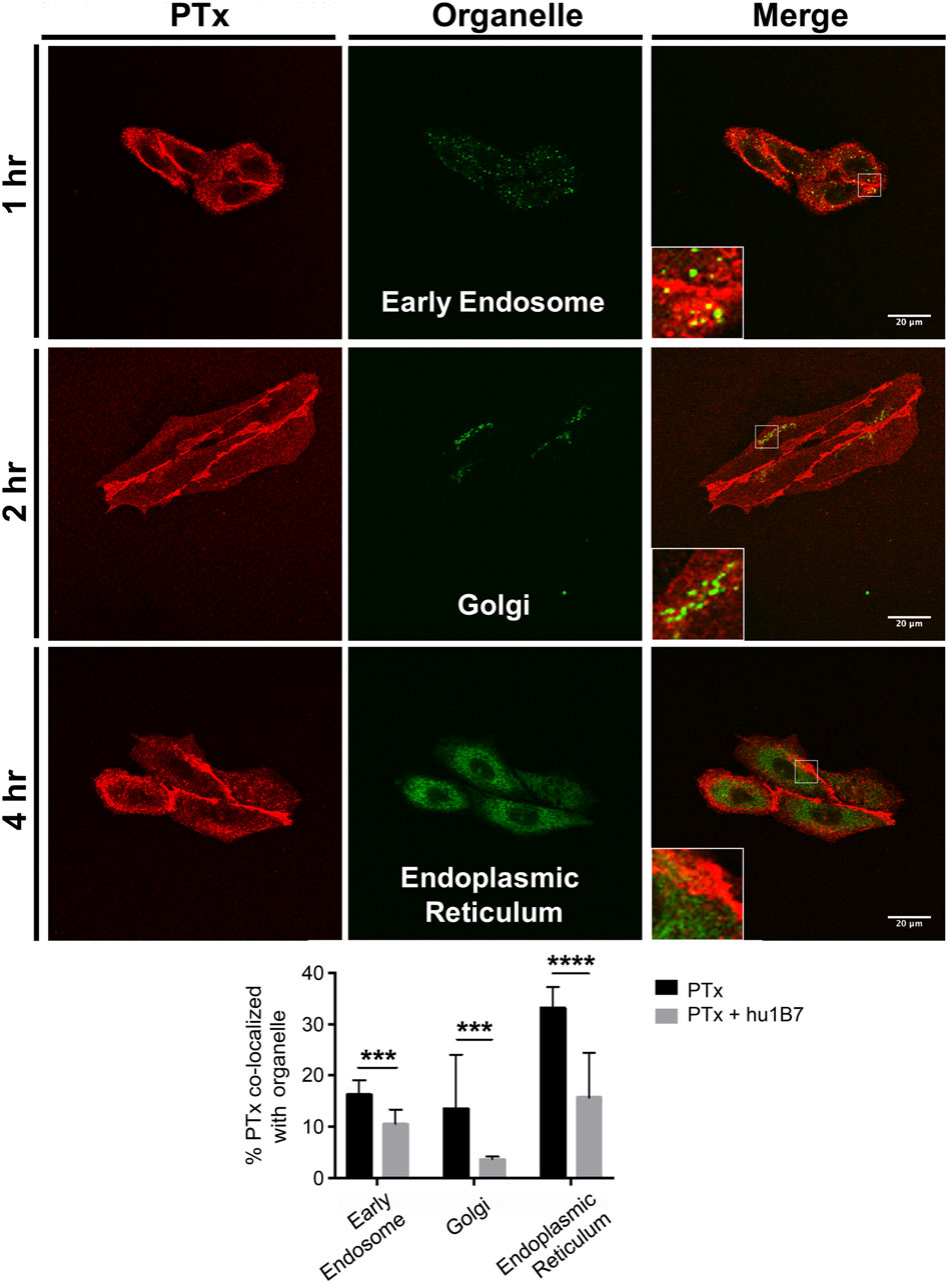
Hu1B7 reduces pertussis toxin (PTx) internalisation into retrograde organelles. CHO cells were grown on cover slides and incubated with 1 nM PTx pre‐equilibrated with a 10,000‐fold molar excess of hu1B7. At the indicated time points, cells were fixed and stained. PTx was detected with a cocktail of mouse anti‐PTx antibodies followed by anti‐mouse IgG‐Cy5 (red), while simultaneously staining for a single organelle (green). CHO cells incubated with PTx for 1 hr were costained with antibodies recognising the EEA1 early endosome marker, whereas those incubated with PTx for 2 hr were costained with antibodies recognising the Golgin‐97, and those incubated with PTx for 4 hr were costained with antibodies recognising the endoplasmic reticulum‐resident protein disulfide isomerase. Each rabbit antibody was followed by goat anti‐rabbit IgG‐AF488. Shown are the mean and standard deviation of at least 30–40 cells from 10 independent images for which the PTx‐only and hu1B7 treatments were performed at the same time, analysed using JACoP plugin for ImageJ. Images are representative of at least three independent experiments; *** p<0.001 and **** p<0.0001. Scale bar, 20 μm

**Figure 8:**
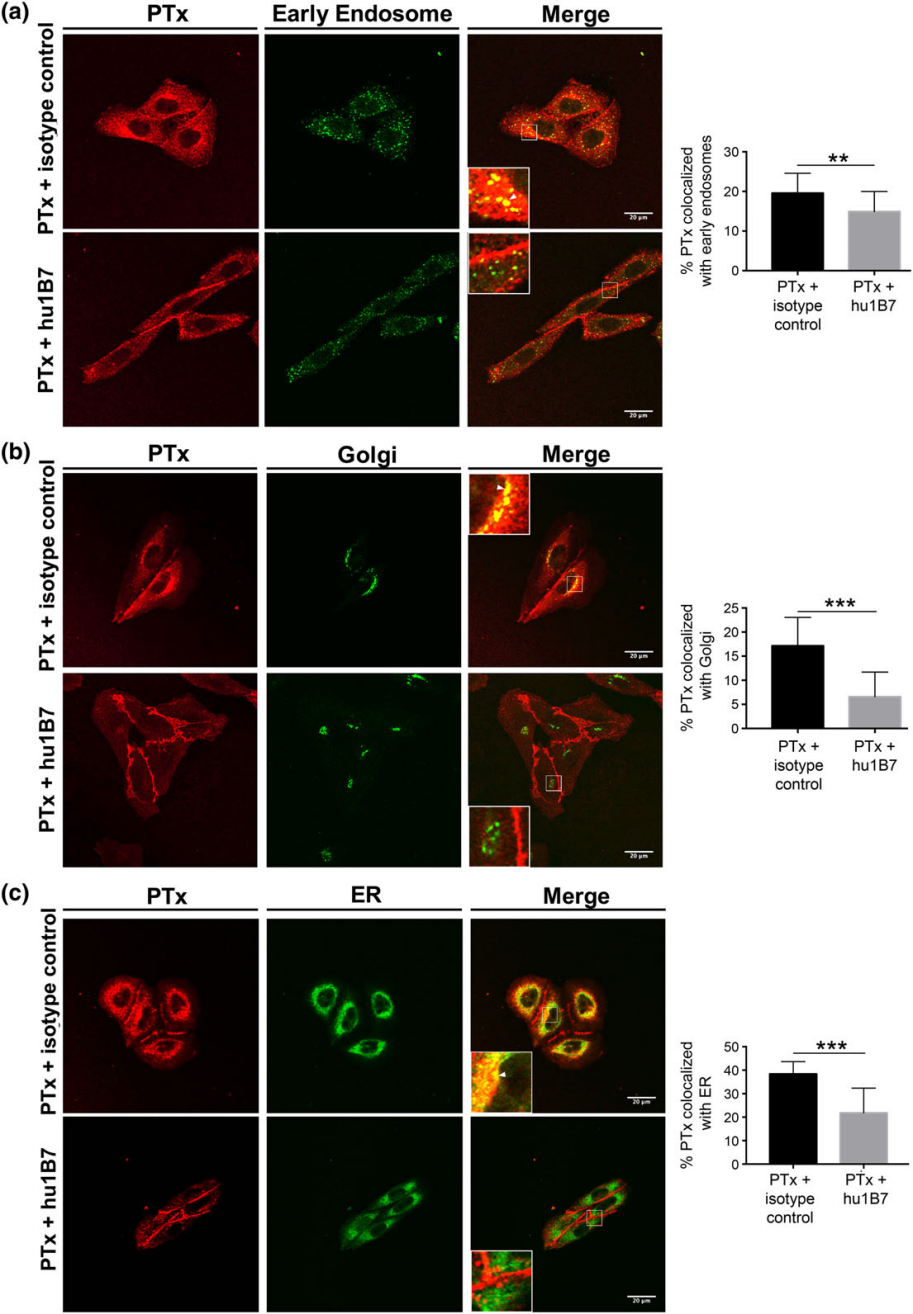
Isotype control antibody does not alter pertussis toxin (PTx) trafficking like hu1B7. CHO cells were grown on cover slides and incubated with 1 nM PTx pre‐equilibrated with a 10,000‐fold molar excess of hu1B7 or human IgG1 isotype control antibody. Cells were stained and data collected and processed as in Figure 7. The percentage of PTx pixels (red) colocalizing with (a) early endosome, (b) Golgi, or (c) endoplasmic reticulum (ER) organelle pixels (green) was analysed with the JACoP plugin for ImageJ. The white arrowhead in the inset indicates colocalization between PTx (red) and organelles (green). Shown are the mean and standard deviation of at least 30–40 cells from 10 independent images with ** p<0.01 and *** p<0.001; hu1B7 and control treatments were performed at the same time to allow comparison. The experiment was performed twice. Scale bar, 20 μm

In contrast, a 10,000‐fold molar excess hu1B7 alters PTx behaviour such that it appears primarily near the CHO cell surface (Figure 7). Qualitatively, in the absence of hu1B7, there is considerable overlap, whereas in the presence of hu1B7, the red and green signals largely segregate. These qualitative observations are supported by quantitative analyses of the images, which supports a significant reduction of PTx colocalization with these three organelles after preincubation with hu1B7 (*P* < 0.001 for all comparisons). Control experiments using PTx preincubated with an isotype control antibody did not exhibit the reduced colocalization with the early endosome, Golgi, and ER that was observed with hu1B7 (Figure 8; *P* < 0.01). Interestingly, hu1B7 did not delay PTx transport into the early endosome, as we observed the same low level of colocalization at 2 and 4 hr for PTx plus hu1B7 but an increase at 1 hr only for PTx preincubated with an isotype control antibody (Figure S2). This suggests that hu1B7 redirects PTx transport at some point prior to its arrival in the early endosome.

When PTx was combined with a 1,000‐fold molar excess m1B7, which does not fully prevent PTx‐mediated CHO cell clustering and is presumed not to block all PTx molecules, PTx and m1B7 were observed to colocalize within cells (Figure S3). A larger molar 10,000‐fold excess of m1B7 or hu1B7 suppresses CHO cell clustering (Wagner et al., 2016). Under these conditions, PTx appears to localize primarily near the cell surface. We therefore speculated that PTx–antibody complexes could be internalised and returned to the cell surface by recycling endosomes but did not observe increased colocalization of PTx–hu1B7 versus PTx alone with the Rab11 marker characteristic of recycling endosomes (<1%, Figure S4). Similarly, we did not observe an increase in PTx–hu1B7 colocalization in the EEA1‐positive early endosomes or the other retrograde organelles (data not shown). We questioned whether hu1B7 could mediate increased transfer of PTx to the lysosome for degradation but again observed very low colocalization levels that were not affected by PTx–hu1B7 coincubation (1–5%, Figure S5). Together, these results support a model in which hu1B7 traps PTx at or near the CHO cell surface by either interfering with endocytosis or with early steps in retrograde transport.

## 3 DISCUSSION

Hu1B7 and hu11E6 are a pair of humanised PTx‐neutralising antibodies that are under evaluation as a potential pertussis therapeutic (Nguyen et al., 2015). A better understanding of the mechanisms by which they neutralise PTx will aid in their development and contribute to our understanding of humoral protection conferred by PTx‐containing vaccines. The neutralising properties of both antibodies rely solely on their abilities to bind secreted PTx, as they do not directly bind *B. pertussis* bacteria or confer complement‐mediated bactericidal activity (Figure 1). Indeed, both appear to act by altering PTx interactions with target cells: hu11E6 by blocking the initial toxin–receptor interaction and hu1B7 by altering toxin internalisation and retrograde trafficking in more subtle ways (Figure 3a).

Although effector functions do not appear to be involved in hu1B7 or hu11E6 neutralisation of PTx toxicity, the interaction of the Fc domain with Fc receptors may increase the protection conferred by antitoxin antibodies through clearance of immune complexes (Ganesan et al., 2012; Montero‐Julian et al., 1995). Each antibody, either by itself or in combination, formed at least small immune complexes with two antibodies and one PTx molecule required to give an ELISA signal (Figure 2a). We anticipate that larger complexes may also be formed through 2:2 binding stoichiometry, because each PTx molecule can bind two antibodies (Figure 2a) and it is reasonable that a single antibody can bind two PTx molecules, based on binding of a bispecific antibody composed of the hu1B7 and hu11E6 binding sites to two PTx molecules (Wagner et al., 2016). The stronger binding of these complexes to FcγRIIb (Figure 2b) suggests that FcγRIIb‐mediated clearance could occur in vivo and may contribute to protection. However, immune complex formation and size depends not only on the stoichiometry of antibody: toxin binding, but also on the antibody and PTx concentrations. Although the antibody concentrations in an animal experiment are easy to determine (e.g., 20‐μg antibody delivered to the ~2‐ml mouse blood volume results in an initial serum concentration of ~10 μg ml^−1^; Wagner et al., 2016), the relevant serum PTx concentration during infection is less apparent, which makes the impact of immune complex formation in vivo harder to assess.

CHO cells are highly susceptible to PTx intoxication and exhibit altered morphology in the presence of very low PTx concentrations, as low as 4 pM (Wagner et al., 2016). For this reason, CHO cells are used in the standard in vitro PTx activity assay (Hewlett et al., 1983) and are an attractive cell line to study the effects of PTx toxicity and antibody neutralisation. Because CHO cells do not express Fc receptors, antibody‐mediated neutralisation of PTx activity in the presence of CHO cells will necessarily omit any role played by Fc receptors on target cells, which may be relevant for PTx intoxication of leukocytes. Other studies addressing the effects of antibodies on toxin neutralisation have also used cell lines lacking Fc receptors (Adekar et al., 2008; Krautz‐Peterson et al., 2008; Yermakova et al., 2016). Our experiments with CHO cells have revealed some key neutralising mechanisms, but understanding the role of the Fc and Fcγ receptors in PTx neutralisation will require further studies, utilising antibody variants with reduced Fcγ receptor affinity such as N297Q agycosylated antibodies or cells from Fcγ receptor‐deficient mice.

To assess how these antibodies may directly neutralise PTx toxicity, we first evaluated their abilities to reduce PTx binding to a model receptor. This is a potent mechanism used by many antibodies to neutralise AB toxins, including three Food and Drug Administration‐approved antibodies targeting anthrax toxin (Raxibacumab and Obiltoxaximab) and the *Clostridium difficile* enterotoxin TcdB (Bezlotoxumab; Wagner & Maynard, 2018). For example, the 14B7 antibody (and Obiltoxaximab precursor) binds an epitope on anthrax toxin that overlaps with that of the cellular receptor and competitively inhibits toxin–receptor binding (Leysath et al., 2009). Similarly, we demonstrated that hu11E6, which binds an epitope near the receptor‐binding site, potently inhibits PTx binding to model receptors in an ELISA (Figure 3b) and to CHO cells, as monitored by immunofluorescence microscopy (Figure 6). By contrast, hu1B7 only partially inhibited PTx–receptor binding in ELISA (Figure 3b), with PTx accumulating near the CHO cell membrane in microscopy studies (Figure 7).

To better understand the fate of hu1B7‐bound PTx on CHO cells, we performed a series of colocalization studies, with analysis of images collected by confocal microscopy. In the absence of antibodies, PTx rapidly undergoes receptor‐mediated endocytosis to enter the retrograde pathway (el Baya, Linnermann, von Olleschik‐Elbheim, & Schmidt, 1997; Plaut & Carbonetti, 2008; Xu & Barbieri, 1996), appearing in endosomes within 30 min and the Golgi and ER after 1 and 2 hr, respectively (Figure 5; Xu & Barbieri, 1995). Strikingly, when PTx was preincubated with an excess of hu1B7, PTx appears primarily near the CHO cell surface, with some molecules present within the cell (Figures 5 and 7). Altered PTx trafficking was not observed with an isotype control antibody (Figure 8).

Together, these results suggest that hu1B7 traps PTx at or near the CHO cell surface either by interfering with endocytosis or other early steps in retrograde transport. Hu1B7 reduces PTx binding to the model receptor fetuin, possibly through a low‐affinity site on the S4 subunit (Figure 3; Sutherland & Maynard, 2009). Although the characterized receptor‐binding site is in PTx subunits S2 and S3, the S4 subunit is proposed to interact with the cell membrane during the penetration step (Montecucco, Tomasi, Schiavo, & Rappuoli, 1986), and the 7F2 antibody recognizing an epitope on the S4 subunit is highly neutralising in vivo and in vitro (Sato, Sato, & Ohishi, 1991). Antibody 1B7 may exert similar behaviour as 7F2 through its weak S4 binding activity.

Antibodies and small molecules have been identified that interfere with toxin intracellular trafficking steps (Gillespie et al., 2013; Krautz‐Peterson et al., 2008; O'Hara & Mantis, 2013; Smith et al., 2009; Stechmann et al., 2010; Yermakova et al., 2016), a function that appears to be shared by hu1B7. These molecules can interfere with toxin transport at the early endosome‐to‐Golgi transfer step, resulting in altered toxin transfer to late endosomes (Yermakova et al., 2016) or recycling endosomes (Krautz‐Peterson et al., 2008). For instance, several antibodies binding the A subunit of ricin appear to delay toxin egress from the early endosomes, leading to toxin accumulation in late endosomes and lysosomes (Yermakova et al., 2016). Interestingly, the epitope of one of these, antibody 1B2, is thought to span the A/B interface (O'Hara & Mantis, 2013) in a similar manner as hu1B7 (Sutherland & Maynard, 2009). Similarly, anti‐ricin antibody Rac18 reroutes the ricin toxin to the lysosomes for degradation (Yermakova et al., 2016), whereas anti‐Shiga Toxin 2 mAbs (Krautz‐Peterson et al., 2008; Smith et al., 2009) and anti‐botulism toxin mAbs (Adekar et al., 2008) can reroute their respective toxins to organelles not involved in normal retrograde transport. Collectively, these data suggest that altered retrograde trafficking is a common mechanism of protection against AB‐ and AB_5_‐type toxins.

The valency and/or size of an immune complex may determine sorting of internalised immunotoxins (van Deurs, Tonnessen, Petersen, Sandvig, & Olsnes, 1986; Yermakova et al., 2016). Internalised immune complexes can be sorted to recycling endosomes and recycled back to the plasma membrane (Krautz‐Peterson et al., 2008) or to lysosomes for degradation (Yermakova et al., 2016). It was recently demonstrated that shiga toxin hitchhikes on the endogenous protein GPP130, which is naturally transported in a retrograde manner (Mukhopadhyay & Linstedt, 2013; Mukhopadhyay, Redler, & Linstedt, 2013). Similarly, it has been suggested that PTx retrograde trafficking requires an intracellular eukaryotic factor, perhaps hitchhiking in a similar manner as shiga toxin (Xu & Barbieri, 1996). If so, m1B7/hu1B7 may interfere with PTx's ability to bind and cotraffic with an endogenous protein.

We sought to further analyse the fate of any internalised hu1B7–PTx complex by costaining for PTx and the Rab‐11 or Lamp‐1 organelles, which are markers of the recycling endosomes and lysosomes, respectively. However, in the presence or absence of hu1B7, we observed no enhanced PTx colocalization with Rab‐11 or Lamp‐1 (Figures S3 and S4), suggesting that these organelles are not involved in the hu1B7‐altered trafficking dynamics of PTx. Although we did not detect the existence of a higher amount of the complex in the Rab‐11^+^ recycling endosomes or the Lamp‐1^+^ lysosomes, other organelles may be involved in the fate of the internalised molecules. For instance, although Rab‐11 labels slowly recycling endosomes, Rab‐4 is considered a marker for fast recycling endosomes (Taguchi, 2013), and Rab‐7 labels late endosomes en route to lysosomes (Vanlandingham & Ceresa, 2009).

Antibodies that neutralise bacterial toxins can be potent therapeutics; indeed, three of the four antibodies approved to treat infectious diseases target toxins by interfering with toxin–receptor interactions, with many more in development (Wagner & Maynard, 2018). The data presented here describe the specific mechanisms utilised by two neutralising anti‐PTx antibodies that protect against disease symptoms in mouse and baboon models of disease. Specifically, the data support reduced retrograde trafficking as a potent mechanism of toxin neutralisation, which appears related to the hu1B7 epitope spanning the S1 and S4 subunits. Additional efforts to identify or engineer antibodies spanning toxin A/B subunit interfaces may hold promise for other diseases.

## 4 EXPERIMENTAL PROCEDURES

### 4.1 Antigens and antibodies

PTx (holotoxin) in glycerol was purchased from List Biological Laboratories, Inc. (Campbell, CA, USA) or obtained through BEI Resources, NIAID, NIH. The expression and purification details for murine m1B7 has been described previously (Sutherland & Maynard, 2009). M11E6 was purified using similar expression and purification procedures. Recombinant, humanised versions (hu1B7 and hu11E6) of m1B7 and m11E6, respectively, with similar affinity and epitope specificity as the parents, were purified as described (Nguyen et al., 2015). Commercial fluorescent secondary antibodies used are indicated.

### 4.2 Antibody binding to whole *Bordetella pertussis*

Antibody binding to *B. pertussis* was analysed by flow cytometry as previously described (Aase et al., 2007). Bacteria were harvested from cultures grown on Bordet Gengou agar, resuspended in PBS, and the OD_600_/ml adjusted to 1. The cells were washed with PBS and incubated with 10 μg of each anti‐PTx antibody or irrelevant antibody in 100‐μl PBS on ice. Bound antibodies were detected after washing with a 1:500 dilution of secondary goat anti‐human‐Fc Alexa Fluor 647. The cells were analysed on a BD SLRII Fortessa flow cytometer (10,000 events per treatment). The cell associated fluorescence for a gated population was determined using the FlowJo_V10 software.

### 4.3 Complement bactericidal assay

The antibody‐dependent complement‐mediated bactericidal assay was performed as described previously (Weiss et al., 2004), with the following modifications. Naïve non‐human primate sera with no detectable anti‐Fha titre was used as a source of complement; similarly, sera from a non‐human primate convalescent for experimental pertussis was used as a positive control. These sera were collected during a previous project approved under IACUC #2016‐00167 (Nguyen et al., 2015). *B. pertussis* strain TohamaI was grown on Bordet Gengou agar plates (Remel, R452432) supplemented with 15% defibrinated sheep's blood (HemoStat Laboratories) at 37°C for 3 days. The bacteria were harvested from the plates and inoculated into 25‐ml Stainer–Scholte media (SSM) to mid‐log phase (an OD_600_ of ~0.22). To reduce expression of the complement resistance phenotype due to expression of the BrkA protein (Barnes & Weiss, 2002), the culture was not allowed to grow for more than 5 hr. This culture was diluted 100‐fold and 5 μl added to duplicate microtitre plate wells containing either 40‐μl SSM or 40‐μl SSM with 1.25 μg antibody. Bacteria and media were mixed gently and equilibrated at 37°C for 30 min before adding 5‐μl baboon sera for a final sera concentration of 10% and a final antibody concentration of 25 μg ml^−1^. The plate was incubated for 1 hr at 37°C; serial dilutions of the bacteria prepared in SSM and three 10‐μl drops per dilution overlaid on Bordet Gengou plates. The plates were incubated at 37°C for 3 days before counting the number of colonies per drop, which is reported as the average of the six drops over the two duplicate samples.

### 4.4 ELISA to determine immune complex formation

An ELISA was used to assess the simultaneous binding of hu1B7 and m11E6 to PTx as previously described (Nguyen et al., 2015). This assay was also used to assess the ability of the hu1B7 and m1B7 or hu11E6 and m11E6 variants to simultaneously bind PTx. Briefly, a humanised antibody was coated on the plate at 1 μg ml^−1^, PTx serially titrated from 500 to 0.005 nM, followed by a murine antibody at 1 μg ml^−1^ and detection with goat anti‐mouse IgG‐HRP at 1 μg ml^−1^. Plates were developed with TMB substrate (Thermo Fisher), quenched with 1N HCl, and the absorbance was measured at 450 nm using a SpectraMax M5 spectrophotometer. The binding curves were fit to the four‐parameter logistic non‐linear curve model using GraphPad Prism 5 to estimate the concentration of each antibody required to achieve 50% maximal binding at saturation (EC_50_).

### 4.5 Antibody binding to FcγRIIb

Binding of antibody alone or after complexation with PTx was determined using an ELISA. ELISA plates were coated overnight at 4°C with human FcγRIIb (2 μg ml^−1^), washed the next day with PBS, 0.05% Tween‐20 (PBST), and then blocked with PBST containing 5% BSA (PBSTB). Serially diluted antibodies (300 to 20 nM antibody) were incubated for 30 min at room temperature with 20 nM PTx in PBSTB for antibody: PTx molar ratios ranging from 15:1 to 1:1. For antibody treatment without PTx, titrations started at 1,000 nM. These mixtures were then added to the FcγRIIb‐coated and blocked wells for 1 hr. To detect bound antibodies, goat anti‐human‐Cκ HRP secondary antibodies were added, with signal that was developed and recorded as above.

### 4.6 PTx–receptor binding ELISAs

To determine whether antibodies can bind preformed PTx–receptor complexes, we adapted a method from Kenimer et al. (1989), as previously described (Acquaye‐Seedah et al., 2018) with fetuin and transferrin as model receptors. High‐binding ELISA plates (Costar) were coated with 10 μg ml^−1^ fetuin (Sigma) in PBS. After overnight incubation at 4°C, the plates were blocked with 150 μl per well blocking buffer (PBS + 5% BSA) at room temperature for 1 hr. Plates were washed three times in wash buffer (PBS, +0.05% Tween‐20) after each incubation step. Serially diluted PTx was then added to the fetuin coat and incubated at room temperature for 1 hr. Antibodies at a concentration of 1 μg ml^−1^ antibody were then added and further incubated for 1 hr at room temperature. Anti‐human‐Fc HRP (Jackson ImmunoResearch) was added at a 1:1,000 dilution in wash buffer. The ELISA plates were developed using the TMB Substrate Kit (Thermo Scientific), the reaction stopped with 1N HCl, and the absorbance read at 450 nm using a SoftMax Pro v5 (Molecular Devices). All plated volumes were 50 μl per well unless indicated otherwise.

The ability of hu1B7 to block PTx binding to fetuin was investigated as previously described (Acquaye‐Seedah et al., 2018). Briefly, fetuin coated overnight at 4°C, at a concentration of 10 μg ml^−1^ in PBS was blocked the next day with PBS–BSA (5%). Serially diluted hu1B7 or hu11E6 antibodies with a constant concentration of 1.6 nM PTx were incubated separately for 30 min at room temperature. The PTx‐Ab complex was then added to the fetuin‐coated plate and incubated further for 1 hr at RT. Bound PTx was detected with a cocktail of six murine anti‐PTx antibodies (10C9, G9A, 3F10, and 7F2, from NIBSC, UK, and m1B7 and m11E6; Sato et al., 1984) each at 0.1 μg ml^−1^. The mouse antibodies were then detected using a goat anti‐mouse antibody HRP‐conjugate, the plate developed, and signal recorded and analysed as above.

### 4.7 PTx retrograde trafficking monitored by immunofluorescence microscopy

Coverslips were seeded with 1 × 10^5^ CHO cells in a six‐well plate and allowed to grow to 50–70% confluency. All samples were washed twice with PBS prior to further processing. The cells were then incubated in blocking buffer (serum and antibiotic‐free Dulbecco's modified Eagle's medium containing 2 mg ml^−1^ BSA) at 37°C with 5% CO_2_ for an additional 30 min. To visualise PTx trafficking, cells were incubated with 100‐μl media containing 1 nM PTx pre‐equilibrated with 10,000 nM hu1B7 or an isotype control for varying times (60, 120, or 240 min) or 10 nM PTx pre‐equilibrated with a 1,000‐fold molar excess of hu11E6 (10,000 nM) or 1 or 10 nM PTx alone (as appropriate for comparison with antibody treatment) for 60 min with at 37°C and 5% CO_2_. At the selected time points, the cells were washed with PBS and fixed with 4% paraformaldehyde for 20 min at room temperature.

After fixation, the cells were washed with PBS three times, permeabilised with PBS containing 0.1% Triton X‐100 for 30 min at room temperature, and subsequently blocked with blocking buffer for 1 hr at 37°C. PTx was detected using a cocktail of mouse monoclonal anti‐PTx antibodies (3F10, G9A, 11E6, 7F2, and 10C9, each at 1 μg ml^−1^) in blocking buffer at 4°C overnight in humid conditions. Simultaneously, individual organelles were visualised with (a) rabbit anti‐EEA1 (early endosome antigen 1) antibody (Thermo Fisher Scientific) specific for the early endosome, (b) rabbit anti‐Golgin‐97 antibodies (Cell Signaling Technology) specific for the Golgi, or (c) rabbit anti‐protein disulfide isomerase antibody (Sigma‐Aldrich) specific for the ER. Next, the cells were washed three times with PBS and incubated with 100 μl of 1:200 The antibody should be Cy5‐conjugated goat anti‐mouse IgG (Invitrogen) and 1:200 Alexa Fluor 488‐conjugated goat anti‐rabbit IgG (Invitrogen) in blocking buffer in the dark at 37°C for 1 hr. After three final washes with PBS, the coverslips were mounted on slides using a drop of 4,6‐diamidino‐2‐phenylindole‐fluoromount‐G (SouthernBiotech).

Images were collected with a Zeiss LSM 710 confocal microscope (Carl Zeiss, Inc.) and processed using the ImageJ software (http://rsbweb.nih.gov/ij). Because there are generally more PTx pixels than organelle pixels in a cell, the frequency of PTx colocalization with an organelle was determined as the $\mathrm{PTx}\;\text{colocalization}\%=\frac{\left(\text{summed intensities of}\;\mathrm{PTx}\;\text{pixels colocalizing with organelle pixels}\right)}{\left(\text{summed intensities of}\;\mathrm{all}\;\mathrm{PTx}\;\text{pixels}\right)}$ within the JACoP plugin for ImageJ (Bolte & Cordelieres, 2006). For experiments with hu11E6, there were very few PTx pixels but many organelle pixels, so we instead calculated colocalization as the $\text{Golgi colocalization}\%=\frac{\left(\text{summed intensities of Golgi pixels colocalizing with}\;\mathrm{PTx}\;\text{pixels}\right)}{\left(\text{summed intensities of}\;\mathrm{all}\;\text{Golgi pixels}\right)}$. Statistical significance was determined with Manders' coefficient, calculated using GraphPad Prism 5 (GraphPad Software, San Diego, CA, USA). Values represent the mean and standard deviation of at least 30–40 cells, with the experiment repeated at least three times.

## AUTHOR CONTRIBUTIONS

E. A. S., Y. H., J. N. S., and J. A. M. planned experiments and analysed results. E. A. S. purified m1B7 and m11E6 antibodies, performed adhesion, cell binding, antibody binding to FcγRIIb, and PTx binding to fetuin assays. Y. H. performed confocal immunofluorescence assays. E. A. S. and J. N. S. performed initial confocal immunofluorescence and receptor binding feasibility studies. J. N. S. performed ELISAs to assess the effects of pH and temperature changes on the hu1B7/PTx‐S1‐220 binding. A. M. D. performed the complement bactericidal assay. J. N. S., E. A. S. and J. A. M. wrote the manuscript with contributions from all authors.

## Supporting information


**Table S1.** Effect of pH/temperature changes on hu1B7/PTx‐S1‐220 binding, measured as a fold‐reduction in ELISA EC_50_.
**Figure S1. PTx can simultaneously bind a model receptor and either the murine m1B7 or m11E6 antibodies.** Transferrin, a glycoslated protein that can serve as a model PTx receptor, was coated on ELISA plates at 2 μg/mL and blocked with PBS + 0.05% Tween‐20 + 4% BSA + 4% FBS. In a separate blocked plate, 4 μg/ml PTx was combined with antibody (m1B7, m11E6 or their respective isotype controls) at the indicated concentrations in blocking buffer with no FBS and allowed to equilibrate at 37°C for 1 hour. Pre‐incubated antibody‐toxin complex was then added to each transferrin coated well and incubated at 4°C overnight. Anti‐mouse‐IgG‐Biotin (MP Biomedicals) was added at a 1/500 dilution in wash buffer and incubated at 37°C for 1 ½ hrs. Next, streptavidin‐HRP (Pierce, Rockford, IL) was added at a 1/8000 dilution in blocking buffer and incubated at 37°C for ½ hr. Signal was developed using the TMB Substrate Kit (Thermo‐Scientific) for ~5 min. The reaction was quenched with 1 N HCl, and the plate read using a SoftMax Pro v5 (Molecular Devices) at 450 nm. Method adapted from (Antoine *et al*., 1990).
**Figure S2. Hu1B7 does not delay PTx internalization kinetics**. PTx colocalization with early endosomes is increased at the one hour time point versus later time points (two and four hours). When PTx is pre‐incubated with hu1B7, no increased colocalization is observed at any of these time points. CHO cells were grown on cover slides and incubated with 1 nM PTx pre‐equilibrated with 10,000‐fold molar excess of hu1B7 or human IgG1 isotype control antibody. At the indicated time points (1 hour, 2 hours and 4 hours), cells were fixed and stained. PTx was detected with a cocktail of mouse anti‐PTx antibodies followed by goat anti‐mouse IgG‐Cy5 (red). Early endosomes were detected with rabbit anti‐EEA1 followed by goat anti‐rabbit IgG‐AF488 (green). For the statistical comparison of PTx + isotype antibody versus PTx + hu1B7 treated CHO cells, 10 independent images, each with 3–4 cells, were collected for each treatment to determine the percent of PTx pixels colocalizing with organelle pixels using JACoP plugin for ImageJ. Shown are the mean and standard deviation comparing colocalization from at least 60–80 cells from two independent experiments.
**Figure S3. Murine antibody m1B7 co‐localizes with PTx in CHO cells.** CHO cells were grown on cover slips to moderate confluency as described in prior figures. Separately, 10 nM PTx was pre‐incubated with a 1,000‐fold molar excess of murine m1B7 antibody in 100 μl DMEM media at 37°C for 30 min and then added to the CHO cells. After two hours, CHO cells were fixed and stained with a cocktail of monoclonal human antibodies specific for PTx (H5, E12, D8 and hu11E6) (Acquaye‐Seedah et al., 2018), followed by goat anti‐human IgG Fc‐AF594 to detect PTx (red). The mouse m1B7 antibody was detected with goat‐anti‐mouse IgG Fc antibody‐AF488 (green). Images were collected as above; 93.7 ± 2.8% of PTx colocalized with murine m1B7 (*n* = 4). Scale bar, 20 μm.
**Figure S4. PTx pre‐equilibrated with hu1B7 does not co‐localize with Rab 11 recycling endosomes.** CHO cells were grown on cover slides to moderate confluency as described in prior figures before adding 1 nM PTx or 1 nM PTx pre‐equilibrated with 10,000 nM hu1B7 in 100 μl media to CHO cells. After one hour, CHO cells were fixed and stained with a cocktail of mouse anti‐PTx antibodies followed by goat anti‐mouse IgG Cy5 to detect PTx (red) and rabbit anti‐Rab11 antibody followed by goat anti‐rabbit IgG‐AF488 (green) to detect recycling endosomes. For the statistical comparison of PTx versus PTx + hu1B7 treated CHO cells, ten independent images, each with 3–4 cells, for each condition were collected to determine the percent of PTx pixels colocalizing with recycling endosomes pixels using JACoP plugin for ImageJ. Shown are the mean and standard deviation of at least 30–40 cells from 10 independent images for experiments performed at the same time. This experiment has been repeated twice each with two technical replicates; NS = not significant. Scale bar, 20 μm.
**Figure S5. PTx alone or pre‐equilibrated with hu1B7 shows low co‐localization with LAMP1 lysosomes.** CHO cells were grown on cover slides to moderate confluency as described in prior figures. CHO cells were pre‐treated with 10 mM or 50 mM NH_4_Cl (an inhibitor of endosome‐lysosome acidification), before adding either 1 nM PTx or 1 nM PTx pre‐equilibrated with 10,000 nM hu1B7. After one hour, CHO cells were fixed and stained with a cocktail of mouse anti‐PTx antibodies followed by goat anti‐mouse IgG‐Cy5 to detect PTx (red) and rabbit anti‐Lamp1 antibody followed by goat anti‐rabbit IgG‐AF488 to detect lysosomes (green). Ten independent images, each with 3–4 cells, for each condition were collected to determine the percent of PTx pixels colocalizing with lysosomes pixels using JACoP plugin for ImageJ. Shown are the mean and standard deviation of at least 30–40 cells from 10 independent images for experiments performed at the same time; NS = not significant. This experiment has been repeated twice. Scale bar, 20 μm.
