# Supplementary material for: Humanised monoclonal antibodies neutralise pertussis toxin by receptor blockade and reduced retrograde trafficking
Source: Cell Microbiol. 2018 Sep 23;20(12):e12948. doi: 10.1111/cmi.12948 (PMC6519169; doi:10.1111/cmi.12948)
Supplement: Supplementary file 1 — Table S1. Effect of pH/temperature changes on hu1B7/PTx‐S1‐220 binding, measured as a fold‐reduction in ELISA EC50. Figure S1. PTx can simultaneously bind a model receptor and either the murine m1B7 or m11E6 antibodies. Transferrin, a glycoslated protein that can serve as a model PTx receptor, was coated on ELISA plates at 2 μg/mL and blocked with PBS + 0.05% Tween‐20 + 4% BSA + 4% FBS. In a separate blocked plate, 4 μg/ml PTx was combined with antibody (m1B7, m11E6 or their respective isotype controls) at the indicated concentrations in blocking buffer with no FBS and allowed to equilibrate at 37°C for 1 hour. Pre‐incubated antibody‐toxin complex was then added to each transferrin coated well and incubated at 4°C overnight. Anti‐mouse‐IgG‐Biotin (MP Biomedicals) was added at a 1/500 dilution in wash buffer and incubated at 37°C for 1 ½ hrs. Next, streptavidin‐HRP (Pierce, Rockford, IL) was added at a 1/8000 dilution in blocking buffer and incubated at 37°C for ½ hr. Signal was developed using the TMB Substrate Kit (Thermo‐Scientific) for ~5 min. The reaction was quenched with 1 N HCl, and the plate read using a SoftMax Pro v5 (Molecular Devices) at 450 nm. Method adapted from (Antoine et al., 1990). Figure S2. Hu1B7 does not delay PTx internalization kinetics. PTx colocalization with early endosomes is increased at the one hour time point versus later time points (two and four hours). When PTx is pre‐incubated with hu1B7, no increased colocalization is observed at any of these time points. CHO cells were grown on cover slides and incubated with 1 nM PTx pre‐equilibrated with 10,000‐fold molar excess of hu1B7 or human IgG1 isotype control antibody. At the indicated time points (1 hour, 2 hours and 4 hours), cells were fixed and stained. PTx was detected with a cocktail of mouse anti‐PTx antibodies followed by goat anti‐mouse IgG‐Cy5 (red). Early endosomes were detected with rabbit anti‐EEA1 followed by goat anti‐rabbit IgG‐AF488 (green). For the statistical comparis [file CMI-20-e12948-s001.docx]

**Supplementary data**

**Table S1.** Effect of pH/ temperature changes on hu1B7/PTx-S1-220 binding, measured as a fold-reduction in ELISA EC_50_.

|  | **Temperature** | | |
| --- | --- | --- | --- |
|  | **25˚C** | **37˚C** | **42˚C** |
| **pH 7.2** | 3 | 4 | 3 |
| **pH 7.0** | 2 | 6 | 3 |
| **pH 6.5** | 2 | 4 | 3 |
| **pH 6.0** | 2 | 4 | 2 |
| **pH 5.5** | 2 | 4 | 4 |
| **pH 5.0** | 2 | 4 | 400 |

**
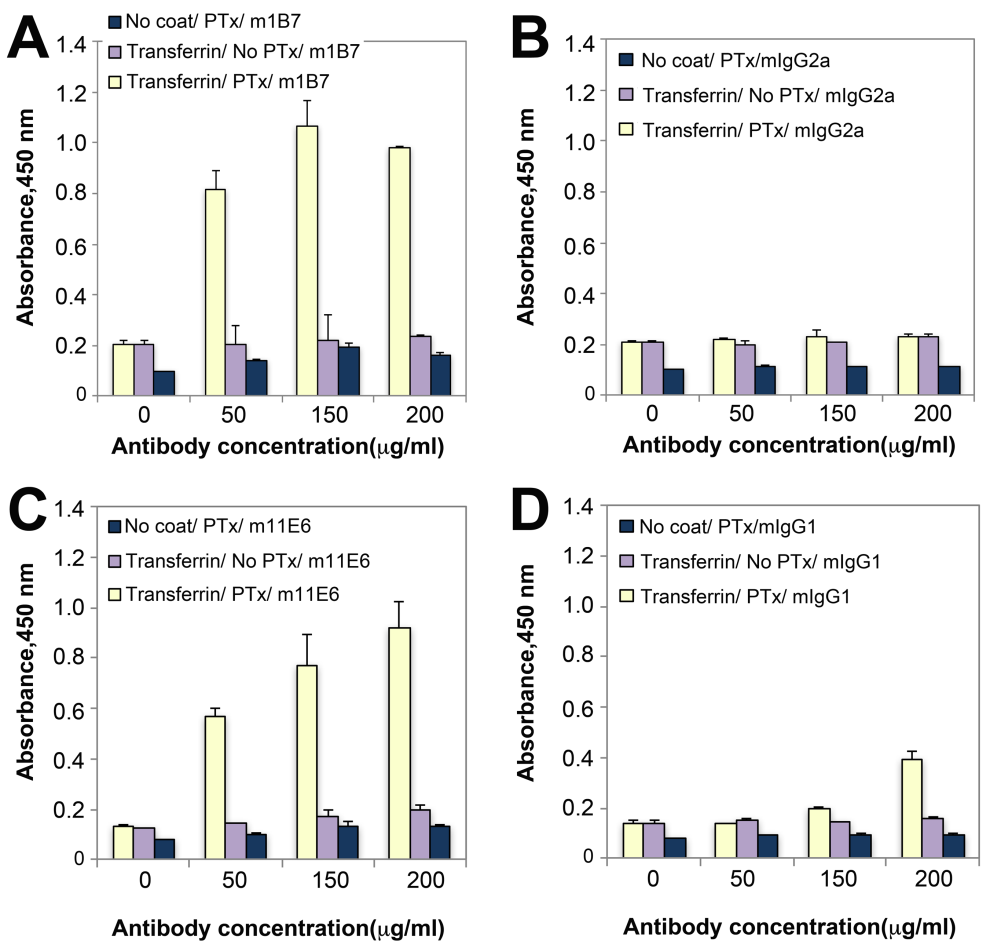
**

**Figure S1. PTx can simultaneously bind a model** **receptor and either the murine m1B7 or m11E6 antibodies.** Transferrin, a glycoslated protein that can serve as a model PTx receptor, was coated on ELISA plates at 2 µg/mL and blocked with PBS + 0.05% Tween-20 + 4% BSA + 4% FBS. In a separate blocked plate, 4 µg/ml PTx was combined with antibody (m1B7, m11E6 or their respective isotype controls) at the indicated concentrations in blocking buffer with no FBS and allowed to equilibrate at 37˚C for 1 hour. Pre-incubated antibody-toxin complex was then added to each transferrin coated well and incubated at 4˚C overnight. Anti-mouse-IgG-Biotin (MP Biomedicals) was added at a 1/500 dilution in wash buffer and incubated at 37˚C for 1 ½ hrs. Next, streptavidin-HRP (Pierce, Rockford, IL) was added at a 1/8000 dilution in blocking buffer and incubated at 37˚C for ½ hr. Signal was developed using the TMB Substrate Kit (Thermo-Scientific) for ~5 min. The reaction was quenched with 1N HCl, and the plate read using a SoftMax Pro v5 (Molecular Devices) at 450 nm. Method adapted from (Antoine *et al.*, 1990).

**Figure S2. Hu1B7 does not delay PTx internalization kinetics**. PTx colocalization with early endosomes is increased at the one hour time point versus later time points (two and four hours). When PTx is pre-incubated with hu1B7, no increased colocalization is observed at any of these time points. CHO cells were grown on cover slides and incubated with 1 nM PTx pre-equilibrated with 10,000-fold molar excess of hu1B7 or human IgG1 isotype control antibody. At the indicated time points (1 hour, 2 hours and 4 hours), cells were fixed and stained. PTx was detected with a cocktail of mouse anti-PTx antibodies followed by goat anti-mouse IgG-Cy5 (red). Early endosomes were detected with rabbit anti-EEA1 followed by goat anti-rabbit IgG-AF488 (green). For the statistical comparison of PTx + isotype antibody versus PTx+hu1B7 treated CHO cells, 10 independent images, each with 3-4 cells, were collected for each treatment to determine the percent of PTx pixels colocalizing with organelle pixels using JACoP plugin for ImageJ. Shown are the mean and standard deviation comparing colocalization from at least 60-80 cells from two independent experiments.

**Figure S3. Murine antibody m1B7 co-localizes with PTx in CHO cells.** CHO cells were grown on cover slips to moderate confluency as described in prior figures. Separately, 10 nM PTx was pre-incubated with a 1,000-fold molar excess of murine m1B7 antibody in 100 µl DMEM media at 37˚C for 30 min and then added to the CHO cells. After two hours, CHO cells were fixed and stained with a cocktail of monoclonal human antibodies specific for PTx (H5, E12, D8 and hu11E6) (Acquaye-Seedah *et al.*, 2018), followed by goat anti-human IgG Fc-AF594 to detect PTx (red). The mouse m1B7 antibody was detected with goat-anti-mouse IgG Fc antibody-AF488 (green). Images were collected as above; 93.7 ± 2.8% of PTx colocalized with murine m1B7 (n=4). Scale bar, 20 μm.

**Figure S4. PTx pre-equilibrated with hu1B7 does not co-localize with Rab 11 recycling endosomes.** CHO cells were grown on cover slides to moderate confluency as described in prior figures before adding 1 nM PTx or 1 nM PTx pre-equilibrated with 10,000 nM hu1B7 in 100 μl media to CHO cells. After one hour, CHO cells were fixed and stained with a cocktail of mouse anti-PTx antibodies followed by goat anti-mouse IgG Cy5 to detect PTx (red) and rabbit anti-Rab11 antibody followed by goat anti-rabbit IgG-AF488 (green) to detect recycling endosomes. For the statistical comparison of PTx versus PTx+hu1B7 treated CHO cells, ten independent images, each with 3-4 cells, for each condition were collected to determine the percent of PTx pixels colocalizing with recycling endosomes pixels using JACoP plugin for ImageJ. Shown are the mean and standard deviation of at least 30-40 cells from 10 independent images for experiments performed at the same time. This experiment has been repeated twice each with two technical replicates; NS = not significant. Scale bar, 20μm.

**Figure S5. PTx alone or pre-equilibrated with hu1B7 shows low co-localization with LAMP1 lysosomes.**  CHO cells were grown on cover slides to moderate confluency as described in prior figures. CHO cells were pre-treated with 10mM or 50mM NH_4_Cl (an inhibitor of endosome-lysosome acidification), before adding either 1nM PTx or 1nM PTx pre-equilibrated with 10,000 nM hu1B7. After one hour, CHO cells were fixed and stained with a cocktail of mouse anti-PTx antibodies followed by goat anti-mouse IgG-Cy5 to detect PTx (red) and rabbit anti-Lamp1 antibody followed by goat anti-rabbit IgG-AF488 to detect lysosomes (green). Ten independent images, each with 3-4 cells, for each condition were collected to determine the percent of PTx pixels colocalizing with lysosomes pixels using JACoP plugin for ImageJ. Shown are the mean and standard deviation of at least 30-40 cells from 10 independent images for experiments performed at the same time; NS = not significant. This experiment has been repeated twice. Scale bar, 20μm.

**References**

Acquaye-Seedah, E., Reczek, E.E., Russell, H.H., DiVenere, A.M., Sandman, S.O., Collins, J.H.*, et al.* (2018). Characterization of Individual Human Antibodies That Bind Pertussis Toxin Stimulated by Acellular Immunization. *Infection and Immunity* **86,** e00004-00018.

Antoine, R. and Locht, C. (1990). Roles of the disulfide bond and the carboxy-terminal region of the S1 subunit in the assembly and biosynthesis of pertussis toxin. *Infect Immun* **58,** 1518-1526.
